# Cisplatin-Induced Rodent Model of Kidney Injury: Characteristics and Challenges

**DOI:** 10.1155/2018/1462802

**Published:** 2018-09-12

**Authors:** Martina Perše, Željka Večerić-Haler

**Affiliations:** ^1^Institute of Pathology, Medical Experimental Centre, Faculty of Medicine, University of Ljubljana, 1000 Ljubljana, Slovenia; ^2^Department of Nephrology, University Medical Centre Ljubljana, 1000 Ljubljana, Slovenia

## Abstract

Cisplatin is an antitumor drug used in the treatment of a wide variety of malignancies. However, its primary dose-limiting side effect is kidney injury, which is a major clinical concern. To help understand mechanisms involved in the development of kidney injury, cisplatin rodent model has been developed. Given the complex pathogenesis of kidney injury, which involves both local events in the kidney and interconnected and interdependent systemic effects in the body, cisplatin rodent model is indispensable in the investigation of underlying mechanisms and potential treatment strategies of both acute and chronic kidney injury. Cisplatin rodent model is well appreciated and widely used model due to its simplicity. It has many similarities to human cisplatin nephrotoxicity, which are mentioned in the paper. In spite of its simplicity and wide applicability, there are also traps that need to be taken into account when using cisplatin model. The present paper is aimed at giving a concise insight into the complex characteristics of cisplatin rodent model and heterogeneity of cisplatin dosage regimens as well as outlining factors that can severely influence the outcome of the model and the study. Challenges for future research are also mentioned.

## 1. Introduction

Cisplatin is an antitumor drug used in the treatment of a wide variety of malignancies (head and neck, lung, testis, ovary, and breast). It has limited use in clinical practice due to its side effects, particularly nephrotoxicity. Nowadays, 20-30% of patients develop acute kidney injury (AKI) after cisplatin treatment despite improvements in therapy [1]. Patients who develop AKI have an increased risk of mortality and are more likely to develop chronic kidney injury (CKI) [2].

To help understand complex mechanisms involved in the development of kidney injury cisplatin rodent model has been developed and extensively used to investigate cisplatin metabolism and the molecular mechanisms of cisplatin nephrotoxicity [3, 4] and to test new generations of platinum-based chemotherapy drugs or adjunctive therapies [5, 6] or other potential agents [7] or strategies to prevent or treat AKI (for instance, stem cells) [8].

Cisplatin rodent model has been recognized as a simple and reproducible model with high clinical relevance [9]. In the past, it was mainly used to investigate acute nephrotoxicity. The investigation of the development of CKI was not gaining any attention, although it was known that cisplatin can have long term effects on the structure and function of the rat kidney after single [10–12] or multiple [13–15] administration. Due to the recent recognition that repeated cisplatin treatment in humans is often associated with renal interstitial fibrosis, leading to CKI [16], and the fact that CKI may develop without being detected [2, 17], the development of better cisplatin mouse models was proposed with aim of increasing the likelihood of identifying novel therapeutic targets for the treatment of cisplatin-induced kidney injury [18].

Namely, cisplatin rodent model has been a subject of a critique as well. Because it has failed to translate treatment strategies of AKI to humans [19] it was argued that it lacks resemblance with human AKI in some aspects, such as morphological changes in the kidney [20] or cisplatin dosing regimen [19]. Thus, it is important to note that cisplatin model has limitations. The main weakness of the model, particularly mouse cisplatin model, is the fact that it is not standardized. This means that almost every laboratory uses its own protocol. Consequently, the protocols among studies differ significantly (i.e., from low nephrotoxic to extremely high lethal doses of cisplatin). The use of wide variety of protocols, thus, causes difficulties in the comparison of results and in the establishment of valid therapeutic strategies. In addition, it also raises the doubt into the usefulness of a model and serious ethical consideration upon animal research. As demonstrated in the paper, rodents, particularly mice, are too frequently exposed to severe suffering. Namely, depending on the cisplatin dosage regimen (cisplatin protocol) rodents may develop not only different severity of nephrotoxicity but also extrarenal toxicity or even systemic toxicity. Thus, for both ethical and scientific reasons a concise insight into complex characteristics of a model in regard to cisplatin dosing regimen is more than needed. Although many excellent articles about cisplatin rodent model have already been published [9, 18, 21], none of them summarized the complexity of its characteristics or discussed the influence of cisplatin dosage regimen on the results or study outcome.

Thus, the first aim of this paper is to briefly summarize the various cisplatin protocols to demonstrate that variations in cisplatin protocols significantly influence not only the results but also the model itself and in some cases may even hamper the comparison and interpretation of the results. The second aim is to point out factors that can significantly influence the model, the outcome of the study, and consequently the validity of the results. The third aim is to point out ethical and scientific concerns and to expose challenges that need to be addressed in the future research.

## 2. Cisplatin Nephrotoxicity in Rodents

The experimental cisplatin-induced nephrotoxicity was first reported in 1971 [22]. Since then numerous studies were published. Over the past years researchers have demonstrated that cisplatin nephrotoxicity is dose dependent and cumulative. Nephrotoxicity can be induced by single or multiple applications of cisplatin. Depending on the dosage, frequency of cisplatin injection, and cumulative dose of cisplatin, animals may develop different severity of acute (early) and chronic (advanced) kidney injury. In rodents cisplatin is usually injected intraperitoneally (ip) and less frequently intravenously (iv) or subcutaneously (sc).

To better understand variations among cisplatin protocols (single/multidosage treatment, low/high nephrotoxic dose, and lethal dose) basic knowledge about pharmacokinetics and underlying mechanisms of cisplatin nephrotoxicity is briefly summarized. However, more information about the pathogenesis of cisplatin nephrotoxicity can be found in many excellent papers [23–28].

### 2.1. The Uptake and Elimination of Cisplatin

Cisplatin is water soluble and low-molecular-weight drug. Following single intraperitoneal administration, cisplatin reaches systemic circulation, where it irreversibly binds to plasma proteins to form inactive complexes, which are considered metabolically inactivated [29]. Unbound cisplatin undergoes distribution to nearly all organs very rapidly. Within 1 hour plasma cisplatin levels decline significantly. Cisplatin is eliminated predominantly by the kidney, much less by biliary (1.2%) [30] and intestinal excretion [31]. The kinetics of cisplatin decay is a biphasic in nature. Cisplatin concentration decreases in the kidney very rapidly after the initial accumulation of the drug (within 15 min) but then again increases and reaches the second peak 48-72 h after a single cisplatin administration [32]. Thereafter it decreases very slowly [33]. Significant levels of the drug were found in the kidney for as long as 1 month [11, 33] or even 3 months after a single nephrotoxic dose of cisplatin [33]. About 43-50% of the drug is eliminated in the urine in the first 24 hours [23, 30], 60-76% in the first 48 hours [23, 34], and about 91% in 72 hours after single cisplatin administration (dose 4-10 mg/kg) [35]. At 72 h after cisplatin administration, the highest concentration of cisplatin was found in the mitochondria (37%), followed by cytosol (27%), nuclei (22%), and microsomes (14%) [32]. Using visualization methodology, it was shown that cisplatin accumulates mostly in the inner cortex and corticomedullary junction of the rat kidney, which is the location of proximal and distal tubules (dose: 5 mg/kg; observation time: day 5). When lethal dose was used (16 mg/kg) cisplatin was found also in renal columns (observation time: day 3), while in the medulla (location of the loop of Henley and collecting tubule) the levels of cisplatin were the lowest, regardless of the dose [36]. In addition, it was found that the intraperitoneal application of cisplatin has a reservoir effect, which prolongs the serum half-life of cisplatin in comparison to the intravenous route [37].

In addition, accumulation and elimination of cisplatin after multidosage treatment has been also examined. It was found that after multiple repeated administration of cisplatin (ip; 5 cycles of 16 mg/m^2^; the elapsed time between each cycle was 21 days) relative cisplatin concentration in the kidney decreased between the first and the second cycle; from the second to the fifth cycle it remained almost constant, while after the fifth cycle it significantly increased [33]. Similarly, another research group observed that multiple repeated administration of cisplatin resulted in decreased renal clearance and increased accumulation of cisplatin in the kidney by each cycle (iv; 3 cycles of 5 mg/kg, a 21-day washout period between each cycle), suggesting long elimination half-life of cisplatin and a collapse of the elimination/detoxification mechanisms [29].

### 2.2. The Pathogenesis of Cisplatin Nephrotoxicity

In rodents cisplatin enters the renal epithelial cells from glomerular filtrate mostly by active transport mediated by the copper transporter 1 (Ctr1) [38], by organic cation transporter 2 (OCT 2), [39, 40], the multidrug and toxin extrusion 1 (MATE1) [41], and to a lesser extent by a passive diffusion (pinocytosis) [36]. In rodents Ctr1 is mainly localized at the basolateral side of both proximal and distal tubules [38], OCT 2 is highly expressed at the basolateral membrane of proximal tubules [39, 40], and MATE1 is localized at the brush-border membrane of proximal tubules [41], while a passive diffusion (pinocytosis) takes place through the cellular membrane (lipid rafts) at the apical membrane of epithelial cells of the proximal tubule [36].

In the renal epithelial cell cisplatin then undergoes metabolic activation to highly reactive molecule, which affects cellular antioxidant system (oxidative and nitrosative status) [4] (demonstrated by decreased superoxide dismutase (SOD), catalase (CAT), glutathione peroxidase (GPx) activity, and decreased glutathione (GSH), glutathione disulfide (GSSG), and nicotinamide adenine dinucleotide phosphate (NADPH) levels) and interacts with different cellular components and macromolecules causing functional and structural damage of proteins (demonstrated by the formation of carbonyl and decrease of P-SH-sulfhydryl proteins), lipids (increased malondialdehyde (MDA), 4-hydroxynonenal- (4-HNE-) oxidative damage) [42], and cellular organelles such as mitochondria [42–44] and endoplasmatic reticulum. Many molecular pathways are triggered in the tubular epithelial cells. Cisplatin nephrotoxicity results in alteration in the number and size of lysosomes and mitochondria [10], disruption of the cytoskeletal integrity and cell polarity, loss of brush border [10, 44], mislocalisation of membrane proteins such as the sodium/potassium ATPase, decreased number of aquaporin water channels (AQP2 and AQP3 in collecting duct and AQP1 in proximal nephron and renal microvasculature) [45], which are responsible for urinary concentration defect [46]. Depending on the dosage cisplatin may lead to cell injury or cell death, i.e., autophagy, apoptosis, and necrosis [47, 48]. In response to cisplatin a number of cytokines are upregulated; various receptors and variety of leukocyte populations are either increased or activated leading to the inflammation. The inflammation of the renal interstitium additionally contributes to the damage [49]. Locally secreted cytokines attract circulating leukocytes into the renal tissue. Erythrocyte accumulation, leukocyte plugging, increased capillary permeability, and leakage of the plasma water into the renal interstitium affect renal function and result in impaired proximal and distal tubular reabsorptive capacity, reversible changes in the renal blood flow, and increased renal vascular resistance (reduced filtration pressure due to afferent arteriolar vasoconstriction was observed 2-3 days after cisplatin administration in rats, 5 mg/kg ip) [49, 50]. Cisplatin has direct effects on the vasculature and glomeruli in rodents. In the kidney vascular injury was reported 1 day after cisplatin administration [51, 52]. Structural changes of the peritubular microvasculature were seen by electron microscopy as endothelial cell swelling, cytoplasmic vacuolization, nuclear degeneration, and detachment [53]. The damage of proximal and distal tubules reduces reabsorptive capacity of the tubular cells, which result in a reduction in glomerular filtration rate (GFR) [54–56], polyuria (reduced reabsorption in tubules due to decreased expression of water channels along the nephron, i.e., aquaporins) [36, 57, 58], a marked defect in urine concentrating ability, increased excretion of proteins [36, 54, 55, 57, 58], glycosuria (urinary glucose wasting) [55], increased excretion of magnesium (Mg) [55], sodium (Na) [57], reduced creatinine (Cr) clearance [55], increased production of hydrogen peroxide, and reduced antioxidant capacity [54]. Extensive morphological damage and functional impairment ultimately lead to the failure of the kidneys to clear nitrogenous wastes from the blood. As a result, blood urea nitrogen (BUN) and uric acid accumulate in the blood (i.e., azotemia denotes elevated levels of nitrogenous waste products in the blood and hyperuricemia denotes excess uric acid in the blood) [59]. Most common metabolites used as biomarkers to diagnose the nephrotoxicity in rodents are BUN and serum Cr, while GFR measurement is less frequent due to technical reasons. Classical GFR measurements involve repeated blood and/or continuous urine sampling over a prolonged time period (5-24 hours), while a novel GFR method involves implantation of transcutaneous device [60].

### 2.3. Molecular Mechanisms and the Inflammation

With use of microarray technology [61–63] it was demonstrated that cisplatin affects numerous genes that are involved in various functions in the kidney, such as biochemical pathways related to creatinine biosynthesis, osmoregulation, kinase signaling, cell cycle-related genes, renal transporters, renal injury, regenerative responses, gene expression changes related to drug metabolism, detoxification, and drug resistance. Researchers have also directed their interest to the investigation of time-associated changes in the kidney gene expression patterns and have showed that there is a wide interplay among numerous genes, whose expression not only depends on cisplatin dosage but also greatly varies during the time course of AKI [61, 64].

In the last two decades experimental models have demonstrated that cisplatin nephrotoxicity is associated with the inflammation and oxidative stress. Numerous studies have been performed to evaluate the role of different immune cells and inflammatory molecules/mediators in cisplatin nephrotoxicity. Plethora of information has been obtained and many controversies emerged. Nevertheless, to elucidate the role of inflammatory cells in the pathogenesis of cisplatin nephrotoxicity, various methods were used, i.e., inhibition of particular cell type using inhibitors or antibodies, generation of mutated mice with specific gene deletion, adoptive transfer of particular inflammatory cell type to mutated mice, or combination of all mentioned methods. Studies report that inhibition of macrophages [52], neutrophils [65–67], or nuclear killer cells [68] does not affect cisplatin nephrotoxicity, while inhibition of both neutrophils and nuclear killer cells results in reduced acute nephrotoxicity [67]. Deletion of either total T cells [69, 70] or CD4^+^ or CD8^+^ T cells [69] or mast cells (Kit^W-sh/W-sh^ mice)[71] attenuates cisplatin nephrotoxicity. The increase in CD4^+^CD25^+^ regulatory T cells (Treg cells) has protective effects as well [69, 70, 72], while the deletion of dendritic cells (CD11c-DTRg mice) worsens cisplatin nephrotoxicity [66, 73] (for more information see Table 1).

In response to renal injury, various inflammatory proteins such as cytokines and intracellular adhesion molecules are produced. Increased expression of various inflammatory proteins (tumor necrosis factor alpha (TNF*α*), intracellular adhesion molecule- (ICAM-) 1, monocyte chemoattractant protein- (MCP-) 1, etc.) or their receptors (TNFR1, TNFR2) was found in the kidney as early as the first day after cisplatin administration [46]. Studies show that the expression of inflammatory mediators differs in a time dependent manner. For instance, interleukin- (IL-) 17A (proinflammatory cytokine) and chemokine (C-X-C motif) ligand- (CXCL-) 1 (neutrophil chemoattractant) peaked at 24 h after cisplatin administration, but 96 h after cisplatin they both returned to the baseline levels. On the other hand, chemokine (C-C motif) ligand- (CCL-) 20 (chemokine for CD4^+^) and CXCL2 (neutrophil chemoattractant) significantly increased not earlier than 96h after cisplatin administration [67]. Renal, circulatory, and urinary levels of various cytokines in acute phase of cisplatin nephrotoxicity are demonstrated in Table 2. Results show that the expression of cytokines differs in accordance with the cisplatin dosage. For instance, nonlethal nephrotoxic dose (10 mg/kg in mice) did not exert significant changes in measured inflammatory proteins in serum or urine (TNF*α* was not increased within first 3 days) [92], while lethal dose (20 mg/kg) exerted significant changes already 24 hours after cisplatin administration (see Table 2).

### 2.4. Histological Characteristics

**Figure 1:**
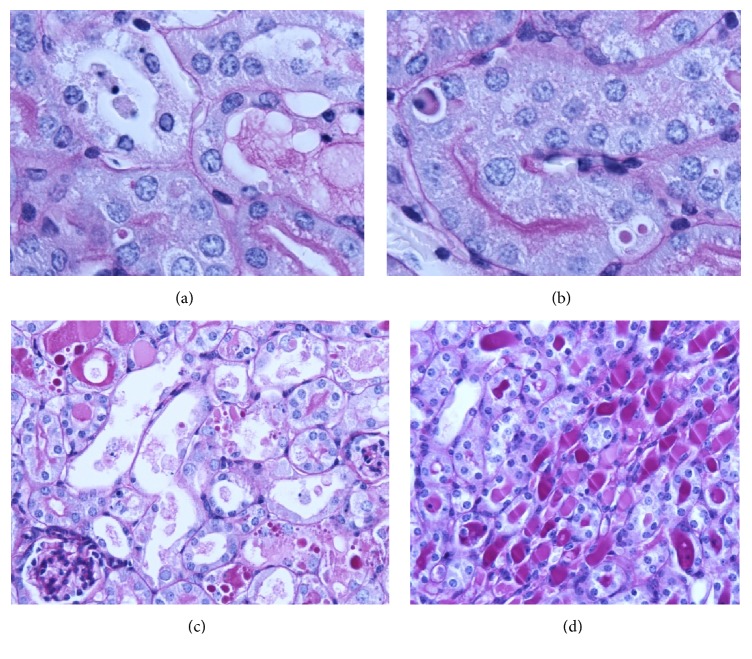
Proximal tubules of BALB/c male mice 4 days after a single cisplatin treatment (17 mg/kg ip): (a,b) hydropic degeneration, pycnotic nuclei, increased cytoplasmic vesicles, cytoplasmic vacuolization, apoptosis, necrosis, loss of brush border, and small hyaline droplets in the cytoplasm (PAS, 400x). (c) Evident loss of brush border, necrosis, desquamation and hyaline/proteinaceous casts, hyaline droplets, and dilated tubules (PAS, 200x). (d) Hyaline casts, hydropic degeneration, and necrosis in distal and collecting tubules (PAS, 200x).

Cisplatin nephropathy in rodents is histologically characterized by degenerative changes in the proximal tubules that consist of hydropic degeneration, pycnotic nuclei, increased cytoplasmic vesicles, cytoplasmic vacuolization, evident loss of the brush border, necrosis and apoptosis of tubular cells, and desquamation of necrotic epithelial cells filling the tubular lumens and forming hyaline casts (see Figure 1) [10].

These morphological changes are more pronounced in the proximal tubules but are also induced in distal and collecting tubules. Studies report wide variability in severity of histological changes ranging from no damage [63] to moderate or severe damage of the kidney [97], as well as reports describing different sites of the damage, i.e., only S3 segments or only S3 segments and distal tubules or throughout the cortex and outer medulla [59] or in the medulla (S3 segments of PT, loops of Henle, and collecting ducts) [4, 36, 98–100]. Mild interstitial inflammation and edema in renal cortex and outer medulla together with some glomeruli exhibiting thickening of the basement membrane were also reported [99]. Nevertheless, studies show that depending on the cisplatin dosage and the time of observation (sampling), histological alterations of AKI can range from no damage to severe damage limited to S3 segment or extended to distal tubules or collecting ducts (see Tables 3 and 4). In contrast, in case of chronic kidney disease repeated administration of low cisplatin dose induced firstly minimal morphological signs of AKI (such as tubules with dispersed heterochromatin and segregated nuclei in epithelial cells) followed by flattened epithelial cells with elongated nuclei. At this time small amount of collagen fibers with mononuclear cell infiltrate can be observed. With repeated administration progressive alterations are induced, such as occasional dilated tubules with mononuclear cells and desquamated epithelial cells. In the recovery period, the size of dilated tubules (lined by flattened and polygonal epithelial cells) gradually diminishes, and atrophic tubules (lined by regenerative cuboidal or cylindrical epithelial cells) appear. Around the affected tubules gradual development of fibrotic areas can be observed. Fibrotic areas are accompanied by infiltration of inflammatory cells such as macrophages and lymphocytes [13, 14]. It was found that the most abundant population of resident leukocytes in the healthy kidney is CD11c^+^MHCII^+^ dendritic cells (renal dendritic cells are CD4^−^ and CD8^−^) [73]. First inflammatory cells that appear in the kidney after cisplatin administration (40 mg/kg) are T cells (CD3^+^), which infiltrate kidney parenchyma within 1 h, reach the peak at 12 h, and decline back on the baseline level by 24 and 72 h. The macrophage and neutrophil infiltration follows later [69]. The reports about the time of increased macrophage or neutrophil infiltration in the kidney vary between 24 h [46, 66], 48 h [52, 70], and 72 h after cisplatin injection [82].

## 3. The Heterogeneity of Cisplatin Protocols

The literature search (PubMed; key words “cisplatin, nephrotoxicity, mice, rats”) has shown a wide variation in doses used for cisplatin-induced nephrotoxicity in both single and multiple dosage protocols. In rats, cisplatin is mostly injected in dose ranging inside nonlethal nephrotoxic range (i.e., between 1 and 8 mg/kg), while in mice a wide variation in cisplatin dose has been used, from low nephrotoxic to highly lethal dose (i.e., between 5 mg/kg and 40 mg/kg).

We have categorized cisplatin protocols according to the extent and severity of cisplatin nephrotoxicity to demonstrate that both the selection of the cisplatin dosage and the time of observation are very important variables that can significantly affect measured parameters and the model. Namely, depending on the cisplatin dosage and time of observation rodents develop acute (early) or chronic (advanced) kidney injury, extrarenal toxicity, or even systemic toxicity. The heterogeneity of protocols and their effects are summarized in Tables 3 and 4.

### 3.1. Acute Nephrotoxicity

According to the definition, AKI is a life-threatening disease that occurs over a period of hours or days as a consequence of septic, ischemic, or toxic insults [102]. However, it is important to note that in case of cisplatin AKI occurs over a period of days in both rodents and humans.

In rodents, acute nephrotoxicity is most frequently induced by a single intraperitoneal injection of cisplatin followed by euthanasia few days later. It can also be induced by multiple applications of low doses of cisplatin for several consecutive days. However, in case of multiple applications, the clinical and histological changes in the kidney develop more slowly than in a single dose treatment.

**A single low nephrotoxic dose of cisplatin** (for instance, 5-8 mg/kg in mice and 1-3 mg/kg in rats) causes mild kidney injuries that can be seen 4-5 days after cisplatin administration histologically (as nuclear pleomorphism (karyomegaly), as mild basophilia and presence of a few necrotic cells), or sometimes as changes in some urine and blood markers (glycosuria, decreased GFR). Importantly, in mice low nephrotoxic dose of cisplatin does not affect serum BUN or Cr levels [44, 55, 56, 103, 104].

**Repeated administration of cisplatin **results in time related increase of many parameters. However, the time course of the disease depends on the dosage, frequency of cisplatin injection, and cumulative dose of cisplatin. For instance, cisplatin treatment in rats (1mg/kg daily for 14 days) resulted in functional renal damage from day 5 onwards. Creatinine increased 2-3-fold from day 5 on, while BUN showed 3-fold on day 5 up to 6-fold by day 14 gradual increase. Glucose was detected in urine from day 5 onwards (150-fold increase on day 5, 18-fold increase on day 7, and 5-fold increase on day 14), without any alterations in serum glucose. Cr clearance decreased to 24-40% on day 5, indicating decreased renal functionality. Histological examination revealed that the incidence and severity of morphological changes increased over time. Minimal severity, such as tubular basophilia with apoptosis in the par recta of proximal tubules (S3), was seen after 3 days of treatment. With increased dosing duration, other degenerative changes were noted, firstly tubular giant cells, crowded basophilic nuclei with prominent nuclei and karyomegaly, and thickened basement membrane in basophilic/degenerative tubules followed by tubular necrosis, hyaline casts and cell debris/exfoliation in the lumen, and tubular dilatation. The distribution of the changes extended from the outer stripe of the outer medulla to the medullary rays (straight tubules, then upper collecting ducts), the cortex (proximal, S2 tubules), and the papilla (loop of Henle, lower collecting ducts) [105].

When using** a single high nephrotoxic dose** (for instance, 10-13 mg/kg in mice and 3-8 mg/kg in rats) 1-2 days after administration only few minimal changes (such as decrease in mitochondria, focal loss of the microvillus brush border, pycnotic nuclei, and increased cytoplasmic vesicles) can be found [10, 44], while morphological changes (such as loss of the brush border, necrotic cells sloughing into the tubular lumen) are usually seen not earlier than 3-4 days after cisplatin administration [10, 44]. Increased BUN/Cr levels are usually observed 3-7 days after cisplatin administration [106–109] and then return to the baseline levels within 14 days [110]. First signs of structural regeneration were observed 7 days after cisplatin injection [10, 111]. However, in case of lethal dose death may occur within 10 days [112].

### 3.2. Chronic Nephrotoxicity

It has been shown that a single nephrotoxic dose of cisplatin not only exerts acute nephrotoxicity but also can have long term effects on the structure and function of the rat kidney [11, 12, 15, 111]. Twenty days after cisplatin injection (5 mg/kg) histological features of chronic nephropathy such as interstitial fibrosis, tubular atrophy, and dilation were found [12]. Gradually developing fibrosis was observed around the affected tubules 14 and 28 days after a single dose of cisplatin (6 mg/kg). Infiltration of macrophages into the injured kidney reached a peak on day 7 and was accompanied by an increase in muscle actin-positive myofibroblasts. On days 14 and 28, the number of macrophages declined, while the number of muscle actin-positive myofibroblasts in the fibrotic area was still high. Cytoplasmic myofilaments were observed in myofibroblasts by electron microscopy [111]. Fifteen months after single cisplatin injection (6 mg/kg) rats had significantly reduced GFR and urinary osmolality and increased number of abnormal proximal tubules (atrophic or hyperplastic), such as presence of glomerular sclerosis and interstitial fibrosis and dilated tubules filled with hyaline casts and lined by simple squamous cells [10, 11]. This indicates that the nephrotoxic effects of cisplatin are long-lasting in rats, like in humans [18]. Unfortunately, we were unable to find any study investigating long term effects of single injection of cisplatin on the structure and function of the mouse kidney.

Nevertheless, chronic nephrotoxicity is usually induced by multiple applications of low doses of cisplatin once a week for a few weeks or once in three-week interval. Few decades ago, Yamate et al. [13] established renal interstitial fibrosis model by administering multiple doses of cisplatin (2 mg/kg once weekly ip for 7 weeks). First mild histological alterations (dispersed heterochromatin and segregated nucleoli) in epithelial cells of the proximal tubules were observed 4 weeks after first injection of cisplatin (at that time BUN and Cr levels were normal), while necrosis or desquamation of renal epithelial cells was seen not earlier than 7 weeks after first injection. At this time BUN and Cr levels increased, tubules were markedly dilated, and regenerative process was observed as well as fibrotic area that developed around affected tubules, accompanied by infiltration of inflammatory cells (macrophages and lymphocytes). Fibrosis was present until the end of the study, i.e., 19 weeks after the last injection of cisplatin, when BUN and Cr finally reached control levels [14].

A large inter- and intraindividual variation was reported in repeated multidose treatment [29, 110]. When cisplatin (4 mg/kg) was repeatedly injected four times at intervals of three weeks (ip, 4 cycles of 4 mg/kg with 21 days of washout period), a decrease in the levels of BUN and Cr was observed after the 2^nd^ injection, but after the 3^rd^ and 4^th^ injection the levels of Cr and BUN increased in an accumulative manner [110]. Finally, it was demonstrated that animals that are recovering from a single injection of cisplatin are less susceptible to a subsequent insult with cisplatin. Ming et al. showed that both the increase in Cr and tubular damage were significantly lower in rats which had received cisplatin (3 mg/kg, ip) 14 days prior to the rechallenge with cisplatin (5 mg/kg, ip) compared with the previously untreated rats. However, attenuation of nephrotoxicity was more obvious in the histological index than in the increase in Cr concentration. Increase in Cr concentration did not correlate with tubular necrosis [113].

### 3.3. Systemic-Extrarenal Toxicity

As demonstrated in Table 3, the lethal dose of cisplatin** (**exceeding LD50) markedly decreases the survival time of animals [108]. For instance, cisplatin in a dose of 20 mg/kg causes severe morphological injuries in mouse kidney and increased BUN levels already 3 days after single intraperitoneal injection, leading to death within 5 days after the injection [84, 128]. Cisplatin in a dose 40 mg/kg causes systemic toxicity within 1-2 days and death within 4 days after single cisplatin injection [44].

In contrast to the nephrotoxic dosage of cisplatin (single or cumulative), where primary injury is located in the kidney, the lethal dosage (single or cumulative) causes systemic toxicity, i.e., injuries in various organs and tissues.

The fact that cisplatin can cause numerous extrarenal injuries is very important issue although it is rarely mentioned in the cisplatin nephrotoxicity research literature. It is important to keep in mind that cisplatin is an antitumor drug that exerts no specific selectivity to certain cell type. Consequently, cisplatin damages not only the dividing cancer cells but also other fast-dividing cells in the body, thus affecting function of many different tissues. Gastrointestinal toxicity, myelosuppression, ototoxicity, neuropathy, nephrotoxicity, and vascular injury (i.e., thrombotic microangiopathy) [129] due to cisplatin treatment are well known side effects in humans [130]. Severe nephrotoxicity, myelosuppression, nausea, and vomiting are particularly dose related effects of cisplatin and occur in up to 30% of patients treated with recommended dosage protocols (with cumulative 50-100 mg cisplatin/m^2^ exposure per cycle) [131].

Extensive signs of cisplatin toxicity can be found in rodents as well [106, 132], like decrease in white blood cells in bone marrow and circulating peripheral blood [106], injuries in gastrointestinal tract [133], testis, lymph tissue, and heart [134], disruption in spermatogenesis [135], systemic endothelial cell injury [52], etc. (for more details see Table 5). It is important to take into consideration the fact that cisplatin causes injuries in various organs in a dose and time dependent manner [106, 132]. Minor injuries were found already after application of nonlethal nephrotoxic dosage of cisplatin. However, when high nephrotoxic dosage is used (which in general severely exceeds LD10 that is by convention the maximal dose used in phase I human studies)[131] toxic effects of cisplatin are more pronounced and when lethal dosage is used (exceeding LD50) systemic injury with multiorgan involvement appears. It is important to note that severe systemic toxicity is accompanied by a generalized host inflammatory response known as the systemic inflammatory response syndrome (SIRS), characterized by intense proinflammatory reaction and release of a cascade of potent inflammatory mediators into the systemic circulation, including TNF-*α*, IL-1*β*, and IL-6 [136], which were regularly observed in the serum and urine of mice treated with lethal dose of cisplatin but not observed when nephrotoxic dose was used (10 mg/kg) (see Table 1). Thus, when using cisplatin animal model, cisplatin dosage and its side effects should be properly included and discussed in the study (i.e., nephrotoxicity versus systemic toxicity).

## 4. Factors Modifying Cisplatin Nephrotoxicity

Various factors can influence the susceptibility, onset, severity, and responsiveness to cisplatin-induced AKI. It is mostly accepted that the onset, severity, and mortality rate of cisplatin nephrotoxicity depend on the cisplatin dosage. However, much less attention is given to other factors such as microbiological state, genetic background, and physical conditions of animals.

### 4.1. Variability among Studies

Susceptibility to cisplatin nephrotoxicity is species specific. Rats are more susceptible to cisplatin toxicity than mice [151]. The rat kidney is also more sensitive to the effects of cisplatin than human kidney [64]. In addition, differences in the susceptibility between strains were also reported (shown in Table 6).

Another factor that can significantly affect cisplatin nephrotoxicity is age. Cisplatin nephrotoxicity was found to be less pronounced in 2-3-week-old unweaned rats compared to 7-8-week-old rats [98, 168, 169]. Younger rats were found to accumulate less cisplatin in their kidneys than older ones [169]. For instance, 6 days after single cisplatin injection (6 mg/kg) 3- and 7-week-old rats had 50 and 30%, respectively, lower concentrations of cisplatin in the kidney than 24-week-old rats [58]. In addition, nephrotoxicity occurred faster in 1-2-week-old rats (6 h after cisplatin) than in 7-week-old rats (3 days after cisplatin). By the time that older rats developed nephrotoxicity, damage in young rats was nearly completely repaired [169, 170]. In addition, differences between neonatal and adult rats were also found in the development of renal interstitial fibrosis. Adult rats developed extensive interstitial fibrosis [111], while in neonatal rats the formation of fibrotic lesions was delayed, and the lesions were limited to the area around the affected nephrons [15]. The reason for differences in cisplatin nephrotoxicity between young and adult rats may be in the stage of kidney development. In contrast with humans and mice, rats nephrogenesis completes 4 to 6 weeks after birth (see Table 7) [153].

In addition, nutritional status of the mother may significantly affect cisplatin nephrotoxicity as well. Offspring of mother rats fed low-protein diets during gestation have lower numbers of nephrons and low renal size until 19 weeks of their age [153].

Similarly to humans [18], aging animals are more susceptible to cisplatin toxicity. Single cisplatin injection (1 mg/kg) resulted in significantly increased BUN and Cr levels and structural degeneration of the proximal tubules in aging rats (52-week-old), beginning 3 days after injection, while in young rats (9-week-old) no significant changes were found (observed period 10 days) [155]. Single dose of cisplatin (16 mg/kg) in aging and young mice resulted in 100% mortality of 24-month-old female mice [149] and 40% mortality of 4-8-week-old mice within 7 days after administration [148, 149]. Increased susceptibility to cisplatin toxicity in aging animals can be ascribed to age-related changes in various tissues, including kidney. Thus, to avoid unwanted toxicity and death in aging animals lower dose of cisplatin should be used.

On the other hand, the influence of sex on cisplatin susceptibility in rodents is less conclusive. Some researchers reported that male Wistar rats have higher susceptibility to cisplatin nephrotoxicity than females [122, 171, 172], while others found no difference [36, 54, 124, 168]. Interestingly, in ovariectomized Wistar rats estrogen showed no effects on cisplatin nephrotoxicity [173], while in castrated Wistar rats testosterone showed protective effects at low dose but harmful effects at high dose [174]. Contradicting results have been reported in mice as well. One study found that male C57BL/6 mice are more susceptible to cisplatin nephrotoxicity than the females [75], while another one found female mice (C57BL/6J and 129Sv) more susceptible [59].

### 4.2. Variability within the Study

Although cisplatin model is reported as reproducible [9], animals do not always respond equally to the same cisplatin protocol (identical dose and administration regimens), even within the same group.

Animals may show enhanced susceptibility [69] or no response [63, 104] to cisplatin toxicity. For instance, in one study 3 of 5 rats developed moderate kidney injury and elevated BUN and Cr levels, while two rats (2/5) had no elevation in the BUN and Cr. Histological assessment revealed mild kidney injury in one rat but no histological alteration in another [63]. According to our experience lethal dose of cisplatin (17 mg/kg) may result in variable response as well. In our case 9/10 mice developed severe AKI, while 1 mouse remained healthy without any increase in BUN values or any clinical signs of illness. Mouse (1/10) experienced only slight drop of body weight (less than 4 %) and remained active, curious, and in good health.

Variability in cisplatin toxicity within the study [69, 98, 104] shows, that there are also other nongenetic factors that play important role in the susceptibility to cisplatin nephrotoxicity or even mortality. Such factors are dietary Mg-depletion [158], reduced intestinal Mg absorption [159], decreased dietary level of selenium [160], hydration status of animals, repeated blood collections during experiment, application of substances, etc. To reduce variability some investigators withhold food and water for few hours prior to cisplatin injection [65]. In addition, circadian timing of cisplatin administration was also shown to have significant effect on BUN, urine volume, urinary concentration of cisplatin, and morphology [133, 161, 175] as well as preventive effect of hydration on cisplatin nephrotoxicity and survival rate when given in lethal dose [152]. Kidney exhibits circadian rhythm of its function in both rodents and humans. The excretion of water and electrolyte and clearance of BUN and Cr from the blood are highly rhythmically regulated within the circadian time scale [176, 177]. In addition, it was shown that IL-6 production is rhythmically regulated, which suggests that cisplatin nephrotoxicity might also depend on kidney sensitivity to diurnal variation in inflammatory reaction without direct cisplatin toxicity [175].

Importantly, it was found that the presence of endotoxin (LPS) increases susceptibility to cisplatin nephrotoxicity, suggesting that coexisting infections might result in synergistic effects and influence cisplatin susceptibility [92, 93]. Since TLR4 receptor was found to be responsible for synergistic effects it was also suggested that acquired or genetic differences in TLR4 signaling or downstream pathways, such as NF-*κ*B activation, might also influence the risk of cisplatin nephrotoxicity [93]. All these results show that microbiological state (latent infection) and genetic background of animals are important factors in cisplatin nephrotoxicity. Unfortunately, both microbiological status and genetic background of the animals are rarely properly reported, which may additionally contribute to confusion and discrepancy of the results.

## 5. Challenges of Cisplatin Rodent Model

The results demonstrate that cisplatin rodent model has many similarities to human nephrotoxicity (Table 8), offering an insight into underlying mechanisms under standardized and controlled conditions in time and dose dependent manner. However, there are also challenges that need to be addressed in the future studies.

### 5.1. A Need for Better Markers

In clinical practice, the diagnosis of kidney injury is mostly based on clinical markers such as BUN and serum Cr, supplemented with data on GFR estimated with different equations, which include additional variables like age, gender, and race [178]. In rodents, the diagnosis of nephrotoxicity is based on determination of BUN and/or Cr in the serum and histological assessment (see Tables 1, 3, and 4). However, the BUN and Cr are not ideal markers and have important deficiencies that need to be taken into consideration. They are unspecific and insensitive in both rodents and humans. The levels of BUN and Cr can be influenced by many other physiological events such as changes in protein synthesis, degradation due to starvation or loss of body weight, gastric or intestinal bleeding, and dehydration [56, 98, 104], all of which are usually seen in cisplatin model (see Table 4) and may contribute to potential underestimation of the actual degree of renal damage. In addition, BUN and Cr lack sensitivity in detecting early stages of kidney injury. As demonstrated in the paper, structural damage within the kidney can be present before BUN or Cr increases. In case of mild form of AKI, BUN and Cr are usually in normal levels. Increase in the BUN and Cr is usually not seen until more than 50 % of the nephrons are functionally damaged in rats and humans, while in mice a rise in the BUN and Cr occurs following the loss of 70-75% of the nephrons, which usually denotes severe nephrotoxicity [55, 56, 104]. Due to the drawbacks of current renal functional parameters, histological assessment is currently the most reliable method to determine the degree of nephrotoxicity in animal research, particularly in mice with mild to moderate AKI.

Nevertheless, since histological evaluation involves invasive procedure and requires experienced pathologist, there is intensive search for more reliable and sensitive biomarkers of nephrotoxicity in both rodents and man [179, 180]. Biomarkers should be noninvasive, indicative of kidney damage, and with ability to detect renal injury before development of marked histological or functional changes [104]. In rodents, collecting blood (for biomarker measurement) during acute phase of kidney injury is dissuaded because it worsens the course of AKI and may result in death of animals. Several candidate biomarkers of AKI in rats have been identified, such as kidney injury molecule-1 (KIM-1), clusterin, and osteopontin [105]. Some of proteins, detected in either urine or blood, were approved for nonclinical drug development. They can be species specific; thus, it is important to stress that they were evaluated only on rats. More information can be found elsewhere [100, 122, 123, 181–183]. Recently, also urinary miRNA as noninvasive biomarker of AKI in humans [184] and rodents [185] was proposed.

### 5.2. The Resemblance to Human Kidney Injury

One oftentimes criticized issue concerning the cisplatin model, particularly mice model, is that morphological changes in the rodents are not equivalent to those observed in human biopsy [20]. It was argued that an acute tubular necrosis in human renal biopsy “*does not accurately reflect the morphological changes in this condition. In essence, ATN (acute tubular necrosis) is the situation in which there is adequate renal perfusion such that there is sufficient blood flow to largely maintain tubular integrity, but not to sustain glomerular filtration*” [20]. Studies report that patients that are suffering from severe AKI in clinical settings [186] show almost normal histological picture or only sporadic mild lesions consisting of degeneration, necrosis, and regenerative changes in the proximal tubule, distal tubule, and collecting ducts [187]. Importantly, in clinical practice, diagnosis of AKI is mostly based on the rise of clinical markers such as BUN and serum Cr and/or the fall in urine output [2]. The renal biopsies are rarely performed in critically ill patients, mainly due to the perceived risk of bleeding complications and general lack of therapeutic consequences. The current knowledge is consequently limited. Animal studies have shown that the morphological changes in the kidney are focally distributed. In human renal biopsy only a small amount of the kidney is captured and thus the histological picture may not be representative. In animals usually the whole size of the kidney is morphologically examined which enables more representative results.

As demonstrated in the paper, depending on the cisplatin protocol rodents may develop various forms of kidney injury, from mild to severe. However, the evaluation of nephrotoxicity with current functional markers is limited particularly in mice, because in mice the BUN and Cr are elevated only when the kidney is already severely damaged. Thus, the critique refers more likely to the use of unspecific markers of nephrotoxicity and to the selection of lethal cisplatin protocols. Therefore, use of better cisplatin rodent models (i.e., use of appropriate cisplatin protocols) is needed to more accurately define the progression of structural and functional changes in the kidney (i.e., from AKI to CKI).

### 5.3. The Use of the Lethal Dose of Cisplatin

Appropriate dosing for cytotoxic anticancer agents in humans has been largely determined upon animal studies in which the goal was to maximize the efficacy and minimize the toxicity, whereas the occurrence of hematologic toxicity (i.e., anemia, leukopenia) usually correlated with proper dose to achieve optimal anticarcinogenic effect [188, 189]. In the 1980s numerous studies on cisplatin acute toxicity alone or in combination with various preventive agents have been reported. It was found that when cisplatin doses were increased above therapeutic doses (the maximum tolerated dose for rats was reported to be 6 mg/kg) the therapeutic effect was reduced due to the toxic side effects of cisplatin [112]. Recently, it was reported that the deletion of CD4^+^ T cell in mice not only did not protect against the kidney injury, but also had harmful effect on the cancer [91]. Thus, to use appropriate cisplatin protocol it is important to consider all side effects of cisplatin (Table 4), including immunosuppressive and carcinogenic ones. These results additionally show that the use of extremely high cisplatin dose (i.e., lethal dose) to produce model to investigate human situation is not scientifically justified and valid.

In addition, since lethal dose induces systemic toxicity, characterized by multiorgan-injuries, the use of lethal dose also raises a question on ethical justification of such experimental protocols. According to severity classification of EU Directive 2010/63, death of animal due to induced illness denotes severe suffering. In accordance with a good laboratory practice, EU legislation and a good science severe suffering of animals in experiments should be avoided. Thus, in case of cisplatin rodent model it is advised to use nonlethal doses of cisplatin or to intervene before severe suffering of animals is manifested.

### 5.4. Clinical Manifestation of The Toxicity

Monitoring and reporting the clinical signs of laboratory animals in experiments are necessary for many reasons such as “*the assessment of animal welfare, compliance with the principle of refinement (e.g., humane endpoints), regulatory compliance (e.g., reporting severity) and, importantly, as a scientific outcome, e.g., in animal models of diseases or safety studies”* [190]. In contrast, we noticed that clinical signs or mortality of animals in cisplatin nephrotoxicity studies are rarely reported. Moreover, we even found statement that* “renal failure per se is not painful”* [191].

However, in cisplatin rodent model clinical signs develop with a delay of a few days and are progressive in nature and dose related. After cisplatin administration, rodents show progressive dehydration and loss of body weight, anemia, and reduced activity. Although rats and mice cannot vomit (nausea and vomiting are major adverse effects of cisplatin therapy in humans) they severely suffer from gastrointestinal malaise already 2 days after low nephrotoxic dose of cisplatin in both mice and rats (6 mg/kg). Gastrointestinal malaise reflects as reduced food and water intake and impaired gastric function (demonstrated by enormously increased gastric content) [137].

In case of the lethal dose of cisplatin (17 mg/kg) clinical signs can be observed not earlier than 2 days after cisplatin injection, when mice show a slight drop of body weight and slight dehydration without other clinical signs of illness. Obvious clinical signs develop progressively on day 3 (obvious dehydration, ruffled hair, and reduced activity), reaching the peak on days 4-5, when gross pancytopenia and significant increase of BUN and Cr in the serum are detected. Mice suffer from hemorrhagic diarrhea and show clinical signs of severe pain (hunched posture, lethargy, orbital tightening, nose bulge, cheek bulge, and changed ear and whisker). Death occurs 4-7 days after cisplatin administration. Before death ataxia with loss of coordination, tremor and rotating body movements after upholding mice by their tails are regularly observed. Systemic injury with multiorgan involvement is reflected by systemic side effects, such as body weight loss, diarrhea, and mortality. At autopsy thymus and splenic involution are found, markedly enlarged stomach filled with food but empty small intestine, both with areas of hemorrhage, similar to what was described by Aggarwal et al. in rats [192]. Thus, to prevent severe suffering of animals it is important to intervene when animals show first signs of severe suffering (see coding of facial expression of pain [193]), are lethargic and moribund, or lose more that 20-25% of body weight.

### 5.5. Challenges in the Investigation of Inflammation

Although numerous studies investigated the role of inflammatory cells in cisplatin nephrotoxicity and demonstrated that various inflammatory cells are implicated in the pathogenesis of cisplatin nephrotoxicity (see Section 2.3), currently the role of inflammatory cells (dendritic cells, T cells, B cells, macrophages, neutrophils, Treg cells, and NK cells) is still difficult to interpret. Lack of surface markers unique to individual type of inflammatory cell population hampers the investigation and limits the interpretation of the results. In addition, the use of surface markers and inhibitors is not always properly reported. Immunology is complex and constantly developing field; thus, results should rely on surface markers and not cell types (i.e., CD11c^+^ instead of dendritic cell).  Table 9 lists recently recognized surface markers expressed on particular inflammatory cells. Additional limitation represents the time and the duration of investigation of inflammatory process in animal studies. The role of inflammatory cells was investigated only in acute state, which is usually in the first three days after cisplatin administration. Since it is known that the role of inflammatory cells in cisplatin nephrotoxicity may differ in response to the phase and severity of the disease, this issue needs to be properly addressed in the future research. For instance, the deletion of CD4^+^ T cells reduced cisplatin nephrotoxicity in acute phase [51, 69], but it had no beneficial effect during disease progression 4 weeks later [91]. Finally, the results are obtained using variety of methods with different degree of sensitivity (morphology, immunohistochemistry, and flow cytometry), which needs to be taken into consideration.

**Figure 2:**
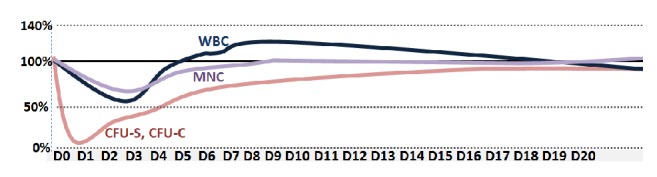
*Cisplatin has immunosuppressive effects* and exhibits cytotoxicity to spleen (CFU-S), granulocyte-macrophage (CFU-C) colony-forming units, and mononuclear cells (MNC) in bone marrow and white blood cells (WBC) in F1 CBAxC57BL female mice (cisplatin: 12 mg/kg, single ip), adapted and modified from Nowrousian et al. [101].

The most important factor that can influence the validity of the results is the fact that depending on the dose cisplatin may exert immunosuppressive effects (Figure 2) [101, 106] or cause systemic toxicity. Therefore, to study inflammatory process in acute or chronic nephrotoxicity the use of appropriate cisplatin dosage regimen reflecting those used in cancer patients is necessary. Otherwise investigation may provide contradictory results, such as in case of anti-inflammatory drugs, where one study reported beneficial effect of COX-2 inhibitor on cisplatin toxicity (cisplatin: 20mg/kg, high lethal dose) [86], while in another study no effect was found (rats (6mg/kg), Wistar Kyoto rats (5 mg/kg, nephrotoxic dose)) [194].

## 6. Conclusions

Cisplatin nephrotoxicity is very complex disease, which involves both complex local events in the kidney and complex interconnected and interdependent systemic effects in the body. Therefore, animal models are indispensable in the investigation of both acute and chronic kidney injury. Although cisplatin rodent model is simple to induce, is not expensive, and has many similarities with human cisplatin nephrotoxicity, it is important to take into consideration that the selection of the cisplatin dosage regimen (cisplatin protocol) significantly affects the characteristics of the model and the outcome of the study. The heterogeneity of cisplatin protocols may contribute to improved knowledge and insight into the development and progression of cisplatin kidney injury, but on the other hand, when used inappropriately, it may hamper the interpretation and usefulness of the results, for instance, when cisplatin dosage results in systemic toxicity instead of nephrotoxicity or when the time frame or method to diagnose mild kidney injury is not optimally selected. Therefore, it is important to recognize that each cisplatin protocol has its own advantages and limitations. The choice of the protocol should thus be based on the scope and aims of a particular study and the characteristics and limitation of a particular cisplatin protocol. However, use of cisplatin protocols that cause acute systemic toxicity should be avoided due to ethical and scientific reasons.

In addition, studies have shown that many factors can affect susceptibility to cisplatin toxicity in rodents. To avoid misinterpretation of the published results, research on animal models should be properly reported in accordance with the ARRIVE guidelines [195] or gold standard publication checklist [196], FELASA recommendations [197, 198], and standardized genetic nomenclature of rodents (http://www.informatics.jax.org/nomen/strains.shtml).

## Figures and Tables

**Table 1:** Genetically altered mice used in the investigation of cisplatin nephrotoxicity.

		**age**						
	**background**	**sex**	**cisplatin**					
**GEM model**	**(breeder)**	**N**	**dose**	**end**	**mortality**	**S**	**measured parameters**	**Ref.**
		25-30g						
	129/SV	male	10 mg/kg				BUN, Cr, Cr clearance, HO-1, p21mRNA	
CYP2e1^−/−^	(NIH)	N=10	ip	D3	nr	↓	and protein, TUNEL	[74]

Oct1^−/−^		8-12wk				ns		
Oct2^−/−^	FVB	male	10 mg/kg			ns		
Oct1/2/^−/−^	(Taconic)	N=11	ip	D3	nr	↓	Histology, Cr, BUN, **ALT↑, ALP↓**	[35]

		6-10wk						
	C57BL/6	male, female	15 mg/kg					
GstP1/P2^−/−^	(UK)	N=5-9	ip	D5	0%	↓	Cr, ATN, WBC	[75]

		11-15wk					D3:BUN, Cr, AST	
Mate1^−/−^		male	15 mg/kg				mortality	
(pyrimethamine)*∗*	C57BL/6	N=6	ip	D6	100%	↑		[41]

Mdk^−/−^	129SV	8-12wk	12 mg/kg	D7	nr	↓	↓BUN, TUNEL, histology, ≈leukocytes	[76]
		male	ip				infiltration (neutrophil, macrophages, Tcells),	
		N=6					Bcl-2, KC protein, MCP-1, MIP-2	

		8-10wk					Survival, histology, BUN, Cr, leukocyte	
		male	15 mg/kg				infiltration, TUNEL, TNF*α*, ICAM1 mRNA,	
PI3K*γ*^−/−^	C57BL/6	N=25	ip	D5	44%	↑	proteins, p-Akt, p-Bad	[77]

		10-12wk						
	B6x129/SF2/J	male	20 mg/kg					
E2F1^−/−^	(Jax)	N=6	ip	D3	nr	↓	↓BUN, Cr, histology,	[78]

p53^−/−^	nr	N=6	20 mg/kg					
(pifithrin-*α*)*∗*	(Jax)		ip	D3	nr	↓	↓BUN, Cr, histology	[79]

p53^−/−^	C57BL/6J	8-10 wk, male	30 mg/kg					
(pifithrin-*α*)*∗*	(Jax)	N=22-29	ip	D3	nr	↓	↓BUN, Cr, histology	[80]

	C57BL/6	8-10wk, male	30 mg/kg					
Bax^−/−^	(Jax)	N=3-8	ip	D3	nr	↓	BUN, Cr, ATN, TUNEL, cytochrome C	[81]

							Histology, BUN, Cr, neutrophils infiltration,	
	C57BL/6	8-10wk, male					TUNEL, Casp-1,-3,-8,-9 activity, IL-1*β*	
Casp-1^−/−^	(Japan)	N=4-15	30 mg/kg	D3	nr	↓		[82]

		8-15 wk, male					BUN, histology, TUNEL	
p21-/-	nr	N=5-6	20 mg/kg	D3	30%	↑		[83]

HO-1^+/-^		12-16wk	20 mg/kg			≈		
HO-1^−/−^	nr	N=8	ip	D3		↑	↓BW, ↑BUN	[84]

	C57BL/10Sn	6-8 wk		D3				
C5^−/−^	(Jax)	male				↓	BUN, Cr, histology, MDA, MPO,	[85]
	BALB/c	N=5-12	20 mg/kg				nitrotyrosine, TNF-*α*, IL-1*β*, ICAM-1, casp-3,8,9, STAT3, NF-*κ*B	
C5aR^−/−^	(Jax)	SPF	ip			↓		

	DBA/1lacJ x	3-4 months					BW, histology, BUN, Cr, Hct,	
mPGES-1^−/−^	C57BL/6 x	male	20 mg/kg				TNF*α*, IL-1*β*;	
(celecoxib)*∗*	129/SV	N=6-16	ip	D3	nr	↓		[86]

TNF*α*^ -/-^		8-9 wk					Histology, BUN, leukocyte infiltration	
(GM6001,	B6129SF2/J	male						
Pentoxifylline)*∗*	(Jax)	N=7	20 mg/kg	D3	nr	↓		[87]

	C57BL/6	8-10wk, male					Histology, BUN, Cr, neutrophil infiltration,	
TLR4^−/−^	(Jax)	N=4-7	20 mg/kg	D3	nr	↓	cytokines	[88]

				D1			Kidney: ↑neutrophils, CXCL1, CXCL2,	
		8-10wk					≈CD4^+^, ↓CD4^+^CD25^+^, Foxp3	
		N=6-10						
TLR9^−/−^	C57BL/6		20 mg/kg	D3	nr	↑	Blood: ↑BUN; Kidney: ↑histology score	[72]

	C57BL/6	8-10 wks	15mg/kg				Blood: ≈BUN; Kidney: ≈histology score	
Tcrd^−/−^	(Jax)	♂, N=9	ip	D4	nr	ns		[67]

Asc^−/−^	C57BL/6	8-10 wk, male	15mg/kg				Blood: ↓BUN;	
TLR2^−/−^	(Jax)	N=5	ip	D4	nr	↓	Kidney: ↓histology score, IL-17A, IL-1*β*	[67]

	C57BL/6	8-10 wk, male	15mg/kg				Blood: ↓BUN; Kidney: ↓histology score	
Ror*γ*t-/-	(Jax)	N=9	ip	D4	nr	↓		[67]

		8-10 wk,		D1			Kidney:↓neutrophils, ≈CD4^+^ T cells, CXCL1,	
IL-17A^−/−^	C57BL/6	male	15mg/kg				CXCL2, CCL2, CCL5	
(anti-IL-17A)*∗*	(Jax)	N=8	ip	D4	nr	↓	Blood: ↓BUN; Kidney: ↓histology score	[67]

	C57BL/6	8-10 wk, male					Histology, BUN, Cr,	
IL-6 ^−/−^	(Jax)	N=4-6	30 mg/kg	D3	nr	ns		[65]

		10-12wk,					↓Mortality,	
	C57BL/6J	male					↑BUN	[89, 90]
IL-6^−/−^	(Jax)	N=9-18	30 mg/kg	D8	47%	↓*∗∗*		

		6-8 wk					Morality, Cr, histology, MPO,	
nu/nu B6.Cg-*Foxn*1^nu^	C57BL/6	male					TNF*α*, IFN-*γ*,IL-1*β*, KC	
(T-cell transfer)*∗*	(Jax)	N=3-14	40 mg/kg	D3	0%	↓		[69]

		6-8wk		D2	6%		Mortality, BUN, Cr, histology, macrophage	
nu/nu (CD4+CD25+Treg	BALB/c	male		D3	31%		infiltration (F4/80 positive cells), TNF*α*, IL-1*β*	
transfer)	(South Korea)	N=16	40 mg/kg	D4	13%	↓		[70]

CD4^−/−^	B6129S2	6-8 wk, male					↓Morality, Cr, histology	
B6.129S2-*Cd4tm1Mak*	(Jax)	N=16	40 mg/kg	D3	38%	↓		[69]

		8-10wk					Blood: ↓Cr; Kidney: ↓histology score	
CD4^−/−^ CD4 tm1Mak	C57BL/6	male						
*GK1.5 monoclonal AB∗*	(Jax)	N=	25 mg/kg	D3	nr	↓		[51]

		8-10wk	10 mg/kg				Blood: ≈BUN, Cr; Kidney: ≈caspase-3,	
CD4^−/−^ CD4 tm1Mak	C57BL/6	male	per week				TUNEL,≈ IL-1, IL-2, IL-4, IL-5, IL-6, IL-10, IL-12, CXCL1, TNF*α*, IFN-*γ*	
*GK1.5 monoclonal AB∗*	(Jax)	N=10	for 4 wks	4wks	nr	ns		[91]

CD8^−/−^	B6129S2	6-8 wk, male					Mortality, Cr, histology	
B6.129S2-*Cd8atm1Mak*	(Jax)	N=16	40 mg/kg	D3	56%	↓		[69]

	C57BL/6	8-10wk, male					Blood: ↓Cr; Kidney:↓histology score,	
CXCR2^−/−^	(Jax)	N=4	25 mg/kg	D3	nr	↓	apoptosis	[51]

	C57BL/6 (wt)	8-10wks	12 mg/kg				Blood: ↓BUN, serum TNF; Kidney:	
Kit^W-sh/W-sh^	(Jax)	N=7	ip	D4	0%	↓	↓histology score	[71]

*∗*A pharmacological inhibitor of particular gene/protein. *∗∗*IL-6 deficiency accelerates the development of nephrotoxicity at the early stage but resists the progress of systemic injury [89].

N: number; S: susceptibility to cisplatin toxicity; ip: intraperitoneally; D: day; ≈: ns; ↓: decrease; ↑: increase; BUN: blood urea nitrogen; Cr: serum creatinine; GFR: glomerular filtration rate; ATN: acute tubular necrosis; BW: body weight; WBC: white blood cells; Hct: hematocrit; TNF*α*: tumor necrosis factor alpha; IL: interleukin; IFN*γ*: interferon gamma; MCP-1: monocyte chemoattractant protein-1; HO-1: heme oxygenase-1; ICAM-1: intracellular adhesion molecule-1; TLR4: toll like receptor 4; COX: cyclooxygenase; Casp3: caspase 3; KC: keratinocyte-derived chemokine; MPO: myeloperoxidase; SOD: superoxide dismutase; CAT: catalase; GSH: glutathione; GPx: glutathione peroxidase; GR: glutathione reductase; GST: glutathione S transferase; MDA: malondialdehyde; ALT: alanine transaminase; AST: aspartate transaminase; ALP: alkaline phosphatase; Cas3: caspase 3; Cas9: caspase 9; CXCL: chemokine (C-X-C motif) ligand; CCL: chemokine (C-C motif) ligand.

**Table 2:** Inflammatory, apoptotic, and oxidative factors in the acute cisplatin nephrotoxicity in rodents.

**tissue/** **method**	**analytes measured**	**D1-D2**	**D3**	**strain**	**Ref.**
				**dose**	
Serum, urine	ELISA: TNF*α*	ns ns	ns ns	C57BL/6,	[92]
Urine/MCA	IL-1*β*, IL-2, IL-4, IL-5, IL-6, IL-9, IL-10, IL-12(p70), IL-17, IFN-*γ*, IP-10, G-CSF, MIP-1*α*, MCP-1, RANTES	ns	n.a.	10wk, male 25-30g 10 mg/kg	
Kidney/RT-PCR	IL-1*β*, TNF*α*, HO-1, IL-18, MCP-1, ICAM-1, TLR4, HMG1	n.a.	ns		
Kidney/ELISA	TNF*α*, MCP-1, MIP-2	n.a.	↑MCP-1, MIP-2		

kidney/RPA	TNF-*α*, TGF-*β*, RANTES, MIP-2, IP-10, MCP-1, TCA3, IL-5, IL-9, IL-15, IFN-*γ*	TNF-*α*, TGF-*β*, RANTES, MIP-2, IP-10, MCP-1, TCA3	TNF-*α*, TGF-*β*, RANTES, MIP-2, IP-10, MCP-1, TCA3	Swiss Webster	[87]
kidney/RT-PCR	TNF-*α*, TGF-*β*, RANTES, MIP-2, MCP-1,ICAM-1, IL-1*ɑ*, IL-1*β*	n.a.	TNF-*α*, TGF-*β*, RANTES, MIP-2, MCP-1, ICAM-1, IL-1*β*	20 mg/kg	

Kidney/RT-PCR	TNF*α*, MCP-1, IL-10	↑ TNF*α*, MCP-1	↑TNF*α*, MCP-1, IL-10	CD-1, Crl Male	[93]
ELISA		↑MCP-1	↑MCP-1	25-35g	
Plasma/ELISA	TNF*α*, MCP-1, IL-10	↑MCP-1	↑ TNF*α*, MCP-1	30 mg/kg	

kidney/RT-PCR	TNF-*α*, IL-6, IL-11, LIF, SOCS3, gp130, OSM, CNTF, CT-1, c-fos, HO-1, Bax, Bcl-xl, Bcl-2, Nrf2, MT-1, MT-2, SOD1, SOD2, COX-2, Casp3	↑TNF-*α*, IL-6, IL-11, LIF, SOCS3, OSM, CNTF, c-fos, HO-1, Bax, Bcl-xl, Bcl-2, Nrf2, MT-1, MT-2	↑TNF-*α*, IL-6, IL-11, LIF, SOCS3, gp130, OSM, CNTF, c-fos, HO-1, Bax, Bcl-xl, Bcl-2, Nrf2, MT-1, MT-2, COX-2, ↓SOD1		
				C57BL/6 Jax 30 mg/kg	[89, 90]
serum/ELISA	IL-6	↑IL-6	↑IL-6		

	**analytes measured**		**D3**		

kidney/RT-PCR	TNF*α*, IL-1*β*, ICAM-1, MCP-1, TGF-*β*1, HO-1		↑TNF-*α*, MCP-1, ICAM-1	C57BL/6 20 mg/kg	[94]
kidney/ELISA	TNF*α*		↑TNF*α*	10-12 wk, male	

serum/ELISA	TNF-*α*		↑TNF-*α*	C57BL/6 male, Jax	
kidney/RT-PCR	TNF-*α*, TNFR1, TNFR2, MCP-1,ICAM-1, HO-1		↑TNF-*α*, TNFR1, TNFR2, MCP-1,ICAM-1, HO-1	20 mg/kg N=4-8; ip	[95]

serum/	TNF-*α*, IL-1*β*, IL-2, IL-6, IL-10, IP-10, MCP-1, RANTES, KC		↑TNF-*α*, IL-1*β*, IL-6, IL-10, IP-10, ↓IL-2		[88]
kidney/			↑TNF-*α*, IL-6, MCP-1, KC, IP-10	C57BL/6 Jax 20 mg/kg	
urine/MCA			↑TNF-*α*, IL-6, IL-2, RANTES, MCP-1, KC, IP-10		

kidney/RT-PCR	TNF-*α*, IL-1*β*, IL-2, IL-6, IL-18, RANTES, MCP-1, ICAM-1, KC, IP-10, TLR4, iNOS, IFN-*ɑ*, IFN-*β*		↑TNF-*α*, IL-1*β*, IL-6, IL-18, MCP-1, ICAM-1, KC, TLR4, iNOS		
kidney/activity	p38MAPK activity, p-JNK phosphorylation - Western		↑p38MAPK, p-JNK		

urine/MCA	IL-1*β*, IL-2, IL-4, IL-5, IL-6, IL-9, IL-10, IL-12 (p70), IL-17, IFN-*γ*, IP-10, G-CSF, TNF-*ɑ*, MIP-1*ɑ*, MCP-1, RANTES		↑TNF-*α*, IL-6, IL-2, RANTES, IP-10	C57BL/6 Jax	[96]
kidney/RT-PCR	HO-1, IL-1*β*, IL-10, TNF-*ɑ*, TNFR1, TNFR2		↑HO-1, IL-1*β*, TNF-*ɑ*, TNFR1, TNFR2	20 mg/kg	
serum/ELISA	TNF-*α*		↑TNF-*α*		

kidney/MCA	TNF-*α*, IL-1*β*, KC, IFN*γ*		↑TNF-*α*, IL-1*β*, KC; ns (24h)	C57BL/6J 40 mg/kg	[69]

kidney/RT-PCR	IL-1*β*, TNF*ɑ*, COX-2, mPGES-1, mPGES-2, cPGES, gp91^phox^, p47^phox^, NOX-1, NOX-3, SOD1, SOD2, SOD3, Bak, Bax, Bcl-2,		↑IL-1*β*, TNF*ɑ*, COX-2, mPGES-1, ↑gp91^phox^, p47^phox^, ↓SOD2, SOD3, ↑Bax, Bak,	DBA/1lacJx C57BL/6 x 129/SV 20 mg/kg	
					[86]

ELISA/serum	TNF-*α*		↑TNF-*α*		
Kidney	PGE_2_, TNF-*α*		↑ PGE_2_, TNF-*α*		

D: day; n.a.: not analyzed; MCA: multiplexed cytokine assay; RPA: ribonuclease protection assay; TNF*α*: tumor necrosis factor alpha; IL: interleukin; IFN*γ*: interferon gamma; MCP-1: monocyte chemoattractant protein-1; HO-1: heme oxygenase-1; ICAM-1: intracellular adhesion molecule-1; TLR4: toll like receptor 4; TGF*β*: tumor growth factor; LIF: leukemia inhibitory factor; CNTF: ciliary neurotrophic factor; gp130: glycoprotein 130; Nrf2: nuclear factor erythroid 2-related factor; OSM: oncostatin M; MT: metallothioneins; SOD: superoxide dismutase; COX: cyclooxygenase; Casp3: caspase 3; iNOS: inducible nitric oxide synthase; KC: keratinocyte-derived chemokine; TNFR1: TNF receptor 1; TNFR2: TNF receptor 2; cPGES: cytosolic prostaglandin E synthase; mPGES: microsomal prostaglandin E synthase; CT-1: cardiotrophin-1; gp91^phox^ and p47^phox^: two major subunits of NADPH oxidase; MIF: macrophage migration inhibitory factor.

**Table 3:** Clinical alterations and the toxicity of cisplatin in mice.

**strain** **(breeder)**	**age** **sex, N**	**R**	**cisplatin dose **	**TP**	**BUN** **mg/dl**	**Cr** **mg/dl**	**end**	**M**	**histology and other comments **	**Ref.**
BALB/c	MTD	iv		D2-4	ns	ns	D10	0%	Histology: proximal tubular epithelial necrosis observed not earlier than D6 - D10; D4: ↓GFR; Urine: ↑proteins, glycosuria,	[55]
	female		7 mg/kg	D6	ns	ns				
	N=3-8			D10	ns	ns				

BALB/c	female	ip	8 mg/kg	D4	ns	ns	D4	0%	Histology: not evaluated D4:↓GFR	[56]
							

C57BL/6J	20-25g	ip	10 mg/kg	D1	24 ±17 **ns**	1.4 ± 0.5 **ns**	D4	0%	Histology: D2 rare changes, D3-D4 evident loss of brush border, pyknotic nuclei, vesicles, Ultrastructure: changes in mitochondria from D1	[44]
Jax	male			D2	44 ±10 **ns**	1.9 ± 0.9 **ns**				
	N=6			D3	105 ±9	12.7 ±1.2				
				D4	228 ± 18	22.4 ±1.8				

C57BL/6 Harlan, UK	male N=6	ip	7.5 mg/kg	D4	1.6x ns	1.2x ns	**D4**	0%	↓BW (4.4±5%); histology: not evaluated	[103]
			**10 mg/kg**	D4	↑4x	↑3.1x	**D4**	0%	↓BW (15.3±11%), histology: moderate ATN: degradation of the brush border, loss of cell-cell adhesion, cellular vacuolation and sloughing of cellular material into lumen	
			**12.5mg/kg**	D4	↑5x	↑3.6x	**D4**	**HEP**	BW (↓27±1%)*∗*, **systemic toxicity**	

C57BL/6	8-12 wk	ip	**15 mg/kg**	D5	n.a.	4.7 ± 0.5	**D5**	**12**%	Histology: all survived mice (7/8) had severe ATN although one had normal Cr levels (6/7); survived mice: ↓BW (19.5%), lack of grooming, lethargy, high morbidity, ↓WBC	[114]
USA	female					(6/7)		**(1/8)**		
	N=8									
								

C57BL/6	male	ip	**15 mg/kg**	D5	↑4x	n.a.	**D5**	**55**%	↓BW (24%), histology: severe ATN: extensive tubular necrosis, loss of the epithelial brush- border, presence of protein casts in the lumen mice began dying on day 3	[115]
Harlan	6-8 wk							**(6/11)**		
	N=11									
								

C57BL/6	11-15wk	ip	**15 mg/kg**	D2	ns	n.a.	**D10**	**100**%	Histology: not evaluated D4:↓BW (20-30%); mice began dying on day 5	[41]
Japan	male			D3	4x	2x				
	N=5-6									

Swiss	Male	ip		D3	↑3x	↑10x	D3	0%	Histology: not evaluated Kidney:↓SOD, CAT, GPx, ↓GSH, ↑MDA	[116]
albino	6-7wks		10 mg/kg							
(India)	N=6									

Swiss	female	ip	12 mg/kg	D3	↑4x	↑4x	D3	0%	Histology: severe ATN; marked necrosis in proximal tubules Kidney:↓SOD, CAT, GPx; ↓GSH, ↑MDA	[117]
albino	6wks									
(India)	N=6									

ICR Thailand	25-30g male N=8	ip	7.5 mg/kg	D3-5	↑ns	ns		0	Preliminary experiment to determine cisplatin dose	[118]
			10 mg/kg	D3 D5	31.7±2.9 ns 53.8 ± 4.2	0.88±0.02 ns 1.74 ± 0.43 ns	D5	0	dose- and time-dependent elevation of BUN and Cr	
			**12.5mg/kg**	D3 D4 D5	52.2 ± 8.3 88.9 ± 18.8 131.8 ± 22	ns ns 2.01 ±0.53	D5	0	15 mg/kg resulted in marked BW loss at d5;	
			15 mg/kg	D3 D4 D5	63.8 ± 17.2 158 ± 77 225 ± 41	ns 2.32 ± 0.59 2.49 ± 0.59	D5	**HEP**	12.5 mg/kg was chosen. Histology: severe ATN: focal necrosis of the proximal tubules throughout the cortex, hyaline casts in tubular lumen, desquamation, and parenchyma degeneration of the tubular epithelium cells	

FVB/n	N=10	ip	4 x 7 mg/kg	D25	ns	ns	D25	0%	Histology: evident loss of brush borders, tubular dilation, less tubular necrosis, evident inflammatory cells and interstitial fibrosis (red Sirius) Urine: ≈KIM-1, ↑NGAL	[119]
Jax			per week							
			4 x 9 mg/kg	D25	ns	ns	D25	90%		
			per week							

**High lethal dose – severe systemic toxicity**										

BALB/c	6-8wk	ip	**25 mg/kg**	D1	ns	ns	D3	0%		[70]
South	male			D2	↑2x	↑2x	D4	80%		
Korea	N=11			D3	↑4x	↑4x	**D5**	**100**%		

BALB/c	6-8wk	ip	40 mg/kg	D1	185 ± 22	2.4 ±0.48	**D1**	**20**%		[70]
South	male						**D2**	**60**%		
Korea	N=21						**D3**	**100**%		

**Nephrotoxicity and antitumor activity in mice with cancer**										

BALB/c	8 wk	ip	11.5 mg/kg	D4	70±42	1.6 ± 0.6	D7	0-12%	Histology: ATN (dose dependent) ***nephrotoxicity and antitumor activity***	[120]
(Harlan)	female		13 mg/kg	D4	130 ± 38	4.5 ± 1.0	D7	0-50%		
	N=8		**14.5 mg/kg**	D4	175±52	5.8 ± 0.7	**D7**	**100**%		
			16 mg/kg	D4	232 ± 40	6.4 ± 1.0	D7	nr		
			19 mg/kg	D4	275 ± 26	6.9 ± 0.2	D7	nr		

C57BL/6	female	iv	7x 1mg/kg every other day	D18	n.a.	n.a.	D18	0/8	Low-dose cisplatin no changes in the BW **Antitumor activity**	[121]
	N=8									
							

C57BL/6	female N=8	iv	7x 5mg/kg every other day	D18	n.a.	n.a.	D18	4/8	High-dose cisplatin 15-30% BW loss, **antitumor activity (experiment was stopped when mice started dying)**	[121]

N: number; M: mortality; R: route of injection; TP: time point; n.a.: not analyzed; ip: intraperitoneally; iv: intravenously; sc: subcutaneously; D: day; ≈: ns; ↓: decrease; ↑: increase; BUN: blood urea nitrogen; Cr: serum creatinine; GFR: glomerular filtration rate; ATN: acute tubular necrosis; HEP: humane endpoint (animals were humanely euthanized due to severe illness). *∗*Animals receiving high dose of cisplatin exhibited systemic toxicity as demonstrated by significant weight loss in excess of 25% of their starting bodyweight, requiring termination of the experiment on day 4 [103].

**Table 4:** Clinical alterations and the toxicity of cisplatin in rats.

**strain ** **(breeder)**	**age **	**R**	** cisplatin** **dose**	**TP**	**BUN** **mg/dl**	**Cr** **mg/dl**	**end**	**M**	**histology and other comments**	**Ref.**
	**sex **									
	**N**									
Han-Wistar (Harlan)	7wks female N=10	ip	2.5 mg/kg	D5 D8 D22	Ns Ns ns	Ns Ns ns	D22	0%	Various doses (0.1; 1; 2.5mg/kg): the effects appeared in a temporal and dose-dependent manner; Histology: D5: mild ATN, D8: minimal ATN, D22: regeneration; majority of alteration seen in the S3 segment of proximal tubules, rarely in distal tubules and collecting ducts	[122, 123]

Han-Wistar (Crl)	7wks Male N=10	ip	3 mg/kg	D2 D3 D5	Ns Ns ↑3.5x	Ns Ns ↑2.5x	D5	0%	Histology: D2,D3,D5: moderate ATN, Urine: ↑leukocytes, ↑ total protein (5.6x)	[104]

Sprague-Dawley (Crl)	6wks Male N=10	ip	3 mg/kg	D2 D3 D5	Ns ↑1.37x ↑2.2x	Ns ↑1.25x ↑2.4x	D5	0%	Histology: D3-D5: minimal to moderate degeneration/necrosis of the S3 segment of proximal tubules in less than half of rats Urine: ↑leukocytes, ↑ total protein (4.6x)	[104]

Wistar WKY (Spain)	7wks female N=4	ip	5 mg/kg	D5	↑2x	↑3.5x	D5	0%	Histology: tubule swelling, loss of brush border membrane, epithelial vacuolization, hyaline casts and cell debris detachment in proximal tubular cells; ↓GFR 3.3x Urine:↑volume (1.9x), proteins (1.9x)	[36, 124]

Wistar ( Spain)	7wks male N=8	ip	5mg/kg	D5	↑2.4x	↑3.3x	D5	0%	Histology: tubular necrosis, swelling and tubular dilatation, extensive epithelial vacuolization, hyaline casts in tubules; ↓BW (8.5%); ↓GFR (4.1x) Urine: ↑Na^+^, ↑volume (2x), ↑proteins (1.7x), ↑urine hydrogen peroxide production, ↓antioxidant capacity in urine	[54]

F344 (NIH)	Male N=7-45	ip	6 mg/kg	D1 D2 D3 D4 D5 D7	Ns ↑1.5x ↑4x ↑3.5x ↑5.3x ↑3.2x	Ns Ns ↑3.7x ↑4.7x ↑4.7x ↑2.6x	D7	0%	↓5 g of BW per day; Histology: D3: focal area of mild ATN in proximal tubules: loss of the brush border, necrotic cells sloughing into the tubular lumen, condensed cytoplasm of many cells, increased number of cytoplasmic vesicles in injured cells; D5: widespread moderate ATN in proximal as and distal tubules: desquamation of necrotic cells, patches of denuded basement membrane, necrotic debris filling the tubular lumens and hyaline casts. Some cells show similar changes as those at D3, lower in height, brush border loss, increased vesicles and swelling; D7: first signs of regeneration, necrotic cells and necrotic debris are still present	[10, 32]

Sprague Dawley (Turkey)	8wk, Male N=6	ip	7 mg/kg	D5	107±5.1	1.4±0.16	D5	0%	Histology: necrosis in proximal tubules, karyomegaly, hyaline casts, desquamation and parenchyma degeneration of tubular epithelial cells, interstitial nephritis; ↓BW (9%); ≈plasma Na^+^ and K^+^ Kidney: ↓GSH, GPx, CAT, ↑MDA	[125]

Wistar (Turkey)	200-250g female N=10	ip	7 mg/kg	D5	↑8x	↑4.5x	D5	0%	Histology: D5: extensive epithelial cell vacuolization, swelling and desquamation Kidney: ↓ SOD, CAT, GPx,↑ MDA, MPO	[126]

Wistar Hanover (Harlan)	male N=5	iv	8 mg/kg	D3 D5	↑2x ↑5.5x	↑1.3x ↑6.5x	D5	0%	Histology: D5: widespread tubular degeneration and regeneration, tubules contained granular and proteinaceous casts, mineralization (5/5) D3: ↑AST (1.2x), urine proteins, glycosuria D5:↓AST, TBIL, GGT, ALP, CI; ↑urine proteins, glycosuria	[64]

Wistar Hanover (Harlan)	male N=5	iv	4x 1 mg/kg	D3 D5	ns ns	ns ns	D5	0%	Histology: D5: similar to 8 mg/kg but less extensive (5/5)	[64]

**Nephrotoxicity and antitumor activity in rats with cancer**										

rats	N=3-5	iv	5mg/kg	D3 D6	51±10 (↑2x) 56±20 (↑2x) /		D6	0%	↓5% BW	[127]


rats	N=3-5	ip	3 x 2.5 mg/kg daily	D3 D6	34±4 (↑0.5x) 73±28 (↑3x)) /		D6	0%	↓17% BW	[127]


rats	N=3-5	ip	7.5 mg/kg	D3 D6	60±10(↑0.2x) 216±82 (↑8x) /		D6	0%	↓22% BW	[127]


N: number; M: mortality; R: route of injection; TP: time point; ip: intraperitoneally; iv: intravenously; sc: subcutaneously; D: day; ≈: ns; ↓: decrease; ↑: increase; BUN: blood urea nitrogen; Cr: serum creatinine; GFR: glomerular filtration rate; ATN: acute tubular necrosis; HEP: humane endpoint (animals were humanely euthanized due to severe illness).

**Table 5:** Cisplatin causes injuries also in other organs and tissues in the body.

**Strain (breeder),**	**cisplatin **		**Other **	**Ref.**
**sex, age, N**	**dose**	**Time**	**comment**	
			**Myelotoxicity**	

CBAxC57BLF1, female, 10-12wk, 20-25g N=3	12 mg/kg ip	D1-23	**gastroenteritis** and body weight loss; ** Femoral bone marrow, blood (WBC, RBC)** more toxic for earlier haemopoietic progenitor cells than for the mature cells. Reduction isseen after 1 day: CFU-S number dropped to 5%, and CFU-C to 6%, BFU-E to0.85%, CFU-E to 60% of the control values on D1; WBC decreased to 53% and MNC to65 % of the control values on D3.	[101]

			**Gastrointestinal toxicity **	

Wistar Male, 200-300g	6 mg/kg ip	D0-2	Day 1: reduction in food and water intake Day 2: ↓food intake (86%),↓water intake(78%), ↑weight of gastric contents (10x)	[137]

C57BL/6 Male, 8-10wk	6 mg/kg ip	D0-2	Day 1: reduction in food and water intake Day 2: ↓food intake (68%),↓water intake(45%), ↑weight of gastric contents (3x)	[137]

B6D2F1 Adult, male 26-30g	8 mg/kg 10 mg/kg 12 mg/kg 14 mg/kg ip	D1,3,6,10,14	**Weight loss** was dose dependent, maximal on day 6 (11- 26%); **reticulocytopenia** was dose dependent with the lowest one on day 6; **necrosis of renal epithelium** (dose dependent) was present by day 10 posttreatment and was still apparent 21-22 days after treatment **azotemia** BUN (dose dependent); dose-dependent** lesions in intestinal epithelium** appeared on days 1-6 (necrosis, hypertrophy, hyperplasia, and macronucleus); **lesions in thecolon** appeared later and were less severe. Mucosal recovery was evident by day 10 posttreatment	[132]

C57BL/6 8-12 wk, 20-25g N=6	27 mg/kg ip	D1-3	Villi in **the small intestine** shortened, reduced number of goblet cells, with increase of apoptosis 3h after cisplatin there were many apoptotic cells in the lamina propria of villi and massive apoptosis of **vascular endothelial cells** in the lamina propria (microvascular endothelial apoptosis) which preceded apoptosis in the epithelium (columnar epithelial cells) Similar results were observed in the **thymus**: massive apoptosis of endothelial cells 3-12h after cisplatin, followed by massive apoptosis of thymocytes 36-72h after cisplatin	[138]

ICR Male, Crl N=5	45 *μ*mol/kg sc	D4	Decrease of total leucocytes, diarrhea observed in all animals	[108]

CBA, UK Male, 6wk N=15	10 mg/kg ip	D10 1,3,5,7,10	Alteration in the kinetics, morphology, and function of the mouse small intestinal mucosa.Severe depression in crypt cell production, evident by 2h and maximal between 12 and 24 h, with only a slow recovery on day 7 and marked depletion in both maltase and sucrose activity in jejunum on days 3 and 5.	[139]

CBA Male, adult N=4	10 mg/kg ip	D1,3,5,7,10	Reduced height of villi and increased height of crypts in small intestine, temporary ablation of crypts, diminished mucosal function (↓disaccharidase activity) observed at D3-10	[140]

Wistar, Adult male N=15	10 mg/kg ip	h1	Mucosal damage, reduced jejunal net fluid and electrolyte absorption (sodium, potassium, chloride)	[141]

Wistar Adult male	6 mg/kg ip	D3	decreased activities of the brush border membrane enzymes (alkaline phosphatase, leucine aminopeptidase, *γ*-glutamyltransferase) in the lining the epithelial cells of intestine, increased production of ROS and alteration in the activities of several antioxidant enzymes (↓SOD, CAT, GPx, GR,G6PD, ↑GST, TR, MDA)	[142]

Wistar Male 7 months	7 mg/kg ip	D5	Changed structure of crypts and villi. Enlarged crypts, reduced length of the villi and epithelium denuded, infiltration of inflammatory cells. In the colon the epithelial tissues and subepithelial layer were affected the most due to cell degeneration, laminin immunopositivity	[143]

			**Hepatotoxicity **	

Wistar female adult	5 mg/kg ip	D5	Histological abnormalities: dispersed area of necrotic hepatocytes, inflammatory cellular infiltration, vacuolation and degeneration of hepatocytes, ↑ ALT, AST, *γ*GT, ALP,↑ total bilirubin in serum; ↓SOD, GSH, GPx, GST, GR,↑MDA and NO, ↑expression of Cas3, Cas9, Bax in liver	[144]

Wistar Male N=15	12 mg/kg ip	D30	↑ ALT, AST, ALP, LDH, *γ*GGT, albumin,↑ total bilirubin in serum ↓SOD, CAT, GSH, GPx, GST, GR,↑MDA, NO, TNF-*α* in liver	[145]

			**Testicular toxicity**	

Sprague-Dawley, male 8wk, 190-250g N=6	7 mg/kg ip	D5 D50	Decreased weight of testes, epididymis, accessory glands (seminal vesicles, prostate), lower sperm concentration, ↓sperm motility, head abnormalities, ↑MDA, GPx, ↓GSH activity in testes	[146, 147]

C57BL/6J Male, 6-16wk, Jax	10 mg/kg ip	D5-31	Changes most evident at D5: majority of tubules were devoid of the late stages of spermatogenesis. Sperm production improved by D12-18 but the decreased number of spermatogenic cells was still evident; spermatocytes and spermatid were TUNEL positive at D14-18. Reduced number of sperm/ml on D31; decreased testis size and weight.	[135]

			**Cardiotoxicity **	

Wistar Male, adult	7 mg/kg ip	D6	↑LDH, CK, TBARS, NO, ↓GSH, ATP degenerative changes and vacuolated cytoplasm of many muscle cells	[134]

			**Ototoxicity **	

CBA/J, female Jax4-8 wk, N=5	14 mg/kg ip	D8	Hearing loss; click evoked auditory brainstem responses threshold elevation at 12± 7 dB	[148, 149]

Wistar Fisher 344	16 mg/kg 3x5 mg/kg ip	D5	Hearing loss, loss of cochlear outer hair cells, sensory and motor nerves conduction velocities alteration; threshold shift of more than 30 dB (at 14kHz measured by auditory evoked brain stem response); 20 dB (at 16 and 24 kHz)	[150]

N: number; D: day; h: hour; ip: intraperitoneally; sc: subcutaneously; WBC: white blood cells; RBC: red blood cells; MNC: mononuclear cells; ↓: decrease; ↑: increase; BUN: blood urea nitrogen; ROS: reactive oxygen species; SOD: superoxide dismutase; CAT: catalase; GSH: glutathione; GPx: glutathione peroxidase; GR: glutathione reductase; GST: glutathione S transferase; G6PD: glucose 6-phosphate dehydrogenase; TR: thioredoxin reductase; MDA: malondialdehyde; TNF*α*: tumor necrosis factor alpha; ALT: alanine transaminase; AST: aspartate transaminase; ALP: alkaline phosphatase; *γ*GT: gamma glutamyl transpeptidase; NO: nitric oxide; Cas3: caspase 3; Cas9: caspase 9; LDH: lactate dehydrogenase; CK: creatine kinase; TBARS: thiobarbituric acid reactive substances; ATP: adenosine triphosphate.

**Table 6:** The acute lethal dose of cisplatin varies among strains of mice and rats.

**Strain (origin),**	**LD100**	**End**	**Ref.**
**sex, age**			
BALB/c (Harlan)	14.5 mg/kg; ip	D7	[109, 120]
female, N=8			

C57BL/6, Japan	15 mg/kg; ip	D10	[41]
Male, 11-15wk; N=5-6			

CBA; female,	16 mg/kg; ip	D7	[149]
24 months, N=3-7			

Wistar rats,	8 mg/kg; ip	D11	[110]
Male, N=17			

	**LD90**		

B6D2F_1_	14 mg/kg; ip	D8	[106]
Male, N=10			

DBA2	16 mg/kg; ip	D10	[112]
Female; N=10			

Swiss Webster	19.5 ± 0.8; iv	D10	[106]
Male, N=10			

	**LD50**		

DBA2	10.7 mg/kg; ip	D10	[112]
Female; N=10	(9.6-11.9mg/kg)		

Swiss Webster	16.0 ± 0.8; iv	D10	[106]
Male, N=10			

NMRI	17.0 mg/kg; ip	D10	[112]
Female; N=10	(14.9-19.7mg/kg)		

Wistar rats	10.8 mg/kg; ip	D10	[112]
Female; N=10	(9.1-12.8 mg/kg)		

Fischer 344 rats	11 mg/kg; ip	D6	[133, 152]
female, 8wks			

N: number; ip: intraperitoneally; iv: intravenously; sc: subcutaneously; D: day; LD: lethal dose; LD100: dose of cisplatin that results in 100% mortality; LD50: dose of cisplatin that results in 50% mortality.

**Table 7:** Comparison of the kidney development among species.

**Glomerular nephrogenesis**			**GFR** **∗**	**Concentrating ability** **∗**
**species**	**onset **	**completion**	**age**	**age**
Human	GD 35-37	35 weeks of gestation	1-2 years	1 year

Rat	GD12.5	Postnatal weeks 4-6	6 wk	6 wk

Mouse	GD11	Before birth	nr	nr

*∗*time when adult levels are reached. GD: gestation day; GFR: glomerular filtration rate. Data from Zoetis et al. [153].

**Table 8:** Examples of the risk factors associated with cisplatin nephrotoxicity in humans and rodents.

**Risk factors**	**rodents**	**humans**
**Race, strain**	Some strains are more susceptible than others (see Tables 3 and 4)	African-Americans have high risk than Caucasians [154]

**Age**	Aging rats and mice are more susceptible [149, 155]	Incidence increases with age [18, 156]

**Hydration**	Hydration reduces nephrotoxicity and mortality [133, 152]	Hydration is used to prevent cisplatin nephrotoxicity [157]

**Mg supplementation**	Increased risk in case of dietary Mg-depletion [158], or reduced intestinal Mg absorption [159], or decreased dietary level of Selenium [160]	Magnesium supplementation is used to prevent cisplatin nephrotoxicity [157]

**Circadian rhythms**	Reduced risk when injected in the middle of the dark period (when the urinary volume is maximal). Difference in survival can be 8-fold and in BUN levels 1.6-fold [152, 161]	?

**Dose **	High doses of cisplatin increase the risk (see Tables 3 and 4)	High doses of cisplatin (↑50 mg/m^2^) increase the risk [28]

**Frequency **	Renal injury is more likely when cisplatin is administered at repetitively close time intervals (daily vs weekly vs 3-week interval).	Renal injury is much more likely when cisplatin is administered at repetitively close time intervals [28]

**Long-term administration**	Nephrotoxicity worsens with the time and repeated long-term treatment [29, 110]	Nephrotoxicity worsens with the time and repeated long-term treatment [18]

**BUN, Cr, GFR**	Unspecific and insensitive A need for better markers	Unspecific and insensitive [17] A need for better markers

**Extra-renal toxicity**	Similar to humans (see Table 5)	Gastrointestinal toxicity, myelosuppression, ototoxicity, neuropathy, nephrotoxicity, vascular injury [130]

**Table 9:** Surface markers of inflammatory cells in cisplatin rodent model.

**Surface marker **	**Inflammatory cells that express surface marker**	**Ref.**
CD45^+^	pan-leukocyte marker	[162]

CD19^+^ (CD79*α*)	marker of B cells	[163]

CD3^+^	marker of T cells	[163]

F4/80^+^	canonical marker of macrophages, monocytes	[73]

CD11c^+^	canonical marker of DC, recently also macrophages, activated CD8+ T cells, plasma B cell blasts, NK	[66, 73, 164]

CD14^+^	monocytes, macrophages, dendritic cells, neutrophils	[165]

CD11b^+^	macrophages, monocytes, neutrophils, dendritic cells	[73]

CD11b^+^CD27^+^	NK (immature, mature)	[166]

CD49b^+^	pan-NK marker, small subset of CD8^+^ T cells, platelets	[68]

F4/80^+^CD11c^−^CD206^+^	macrophages M2	[167]

F4/80^+^CD11c^+^	macrophages M1	[167]

F4/80^−^CD11c^+^	dendritic cells	[167]

GR-1^+^	neutrophils, monocytes	[73]

GR-1^+^ CD11b^+^ CD11c^+^	monocyte-derived proinflammatory DC	[73]

GR-1^+^ CD11b^+^ CD11c^−^	neutrophils, monocytes	[73]

7/4^+^	monocytes, neutrophils	[73]

Ly6G^+^	neutrophils	[73]

7/4^+^Ly6G^−^	monocytes	[73]

DC: dendritic cells; NK: natural killer cells

## Abbreviations

4-HNE: 4-Hydroxynonenal
AKI: Acute kidney injury
ALP: Alkaline phosphatase
ALT: Alanine transaminase
AST: Aspartate transaminase
ATN: Acute tubular necrosis
ATP: Adenosine triphosphate
BUN: Blood urea nitrogen
Casp: Caspase
CAT: Catalase
CFU-C: Granulocyte-macrophage colony-forming unit
CFU-S: Spleen colony-forming unit
CK: Creatine kinase
CKI: Chronic kidney disease
CNTF: Ciliary neurotrophic factor
COX: Cyclooxygenase
cPGES: Cytosolic prostaglandin E synthase
Cr: Creatinine
CT-1: Cardiotrophin-1
Ctr 1: Copper transporter 1
G6PD: Glucose 6-phosphate dehydrogenase
GFR: Glomerular filtration rate
gp130: Glycoprotein 130
GPx: Glutathione peroxidase
GR: Glutathione reductase
GSH: Glutathione
GSSG: Glutathione disulfide
GST: Glutathione S transferase
HEP: Humane endpoint
HO-1: Heme oxygenase-1
ICAM-1: Intracellular adhesion molecule-1
IFN*γ*: Interferon gamma
IL: Interleukin
iNOS: Inducible nitric oxide synthase
ip: Intraperitoneally
iv: Intravenously
KC: Keratinocyte-derived chemokine
KIM-1: Kidney injury molecule-1
LDH: Lactate dehydrogenase
LIF: Leukemia inhibitory factor
MATE1: Multidrug and toxin extrusion 1
MCA: Multiplexed cytokine assay
MCP-1: Monocyte chemoattractant protein-1
MDA: Malondialdehyde
MIF: Macrophage migration inhibitory factor
MNC: Mononuclear cells
mPGES: Microsomal prostaglandin E synthase
MPO: Myeloperoxidase
MT: Metallothioneins
NADPH: Nicotinamide adenine dinucleotide phosphate
NO: Nitric oxide
Nrf2: Nuclear factor erythroid 2-related factor
OCT 2: Organic cation transporter 2
OSM: Oncostatin M
PGE: Prostaglandin E
ROS: Reactive oxygen species
RPA: Ribonuclease protection assay
sc: Subcutaneously
SOD: Superoxide dismutase
TBARS: Thiobarbituric acid reactive substances
TGF*β*: Tumor growth factor
TLR4: Toll like receptor 4
TNFR1: TNF receptor 1
TNFR2: TNF receptor 2
TNF*α*: Tumor necrosis factor alpha
TR: Thioredoxin reductase
WBC: White blood cells
*γ*GT: Gamma glutamyl transpeptidase.
